# Utility of Coffee in Soldiers’ Diet

**Published:** 1862-07

**Authors:** 


Utility of Coffee in Soldiers' Diet.—M. Larrey, having been called upon by the Council of Health of the Department of the Seine for his opinion as to the desirableness of extensive employment of coffee in the soldier's diet, speaks in his reply in the warmest terms concerning its use. He states that upon his father's recommendation it was employed freely in Algeria, with the best effects upon the soldier's health, as a substitute for brandy. It has also seemed to act in some measure as a prophylactic to intermittent fever.—*London Med. Times and Gazette.*

